# Exploring the relationships between epistemic beliefs about medicine and approaches to learning medicine: a structural equation modeling analysis

**DOI:** 10.1186/s12909-016-0707-0

**Published:** 2016-07-18

**Authors:** Yen-Lin Chiu, Jyh-Chong Liang, Cheng-Yen Hou, Chin-Chung Tsai

**Affiliations:** Graduate Institute of Digital Learning and Education, National Taiwan University of Science and Technology, #43, Sec. 4, Keelung Rd., Taipei, 106 Taiwan; Graduate Institute of Applied Science and Technology, National Taiwan University of Science and Technology, #43, Sec. 4, Keelung Rd., Taipei, 106 Taiwan

**Keywords:** Epistemic beliefs, Approach to learning, Learning medicine, Medical students

## Abstract

**Background:**

Students’ epistemic beliefs may vary in different domains; therefore, it may be beneficial for medical educators to better understand medical students’ epistemic beliefs regarding medicine. Understanding how medical students are aware of medical knowledge and how they learn medicine is a critical issue of medical education. The main purposes of this study were to investigate medical students’ epistemic beliefs relating to medical knowledge, and to examine their relationships with students’ approaches to learning medicine.

**Methods:**

A total of 340 undergraduate medical students from 9 medical colleges in Taiwan were surveyed with the Medical-Specific Epistemic Beliefs (MSEB) questionnaire (i.e., multi-source, uncertainty, development, justification) and the Approach to Learning Medicine (ALM) questionnaire (i.e., surface motive, surface strategy, deep motive, and deep strategy). By employing the structural equation modeling technique, the confirmatory factor analysis and path analysis were conducted to validate the questionnaires and explore the structural relations between these two constructs.

**Results:**

It was indicated that medical students with multi-source beliefs who were suspicious of medical knowledge transmitted from authorities were less likely to possess a surface motive and deep strategies. Students with beliefs regarding uncertain medical knowledge tended to utilize flexible approaches, that is, they were inclined to possess a surface motive but adopt deep strategies. Students with beliefs relating to justifying medical knowledge were more likely to have mixed motives (both surface and deep motives) and mixed strategies (both surface and deep strategies). However, epistemic beliefs regarding development did not have significant relations with approaches to learning.

**Conclusions:**

Unexpectedly, it was found that medical students with sophisticated epistemic beliefs (e.g., suspecting knowledge from medical experts) did not necessarily engage in deep approaches to learning medicine. Instead of a deep approach, medical students with sophisticated epistemic beliefs in uncertain and justifying medical knowledge intended to employ a flexible approach and a mixed approach, respectively.

## Background

Understanding how medical students are aware of medical knowledge and how they learn medicine is a critical issue of medical education. Students’ epistemic beliefs (i.e., beliefs in relation to the nature of knowledge and knowing) are regarded as playing an influential role in their learning approaches, including motivation and strategies [1, 2]. An extensive body of recent research has examined the relationships between students’ epistemic beliefs and their approaches to learning e.g., [3–6]. As Hofer [7] indicated, students’ epistemic beliefs may vary in different domains. Pintrich [8] proposed that epistemic thinking is domain-specific, and suggested that future research examine the domain specificity of epistemic beliefs. A growing number of studies have focused on examining domain-specific epistemic beliefs such as epistemic views on general science [9, 10] and specific disciplines in science including physics and biology [6, 11]. Therefore, it may be beneficial for medical educators to better understand medical students’ epistemic beliefs regarding medicine.

In order to raise reflective and self-directed medical practitioners, over the past decades, medical education has focused on curricular change to encourage medical students’ deep understanding, critical thinking, and problem-based learning [12–14]. Consequently, medical educators as well as researchers have paid attention to medical students’ approaches to learning and have become interested in the relations between approaches to learning and academic outcomes and clinical success [12, 14]. As a result, the main purpose of this study was to investigate medical students’ medical-specific epistemic beliefs, approaches to learning medicine, and the relationships between them.

Epistemic beliefs could be conceptualized as a system of beliefs with dimensions relating to the nature of knowledge and knowledge acquisitions [15]. Hofer and Pintrich further recommend that epistemic beliefs can be identified as the nature of knowledge with dimensions regarding certainty of knowledge (knowledge is fixed or uncertain) and simplicity of knowledge (knowledge is absolute or relative); and the nature of knowing with dimensions containing source of knowledge (knowledge from authority can be trusted or be challenged) and justification for knowing (knowledge claims should be accepted or justified) [2]. Each dimension can be viewed as a polarized continuum from naive perspectives (e.g., knowledge is certain, stable and absolute) to sophisticated perspectives (e.g., knowledge is tentative, complex and relative) [2, 16]. With respect to the domain-specific epistemic beliefs, science-specific epistemic beliefs can be conceptualized as dimensions including source (e.g., scientific knowledge transmitted from experts), certainty (e.g., scientific solution is certain), development (e.g., scientific knowledge is evolving), and justification (scientific knowledge should be evidenced) [10].

Approaches to learning refer to students’ ways of performing their academic tasks, by which the learning outcomes may be affected [17]. The defining features of approaches to learning are recognized as deep and surface approaches [18, 19]. While a deep approach is denoted as meaningful learning, a surface approach is inferred to mean rote learning [20]. A deep approach indicates active engagement with the learning contents, a holistic style (relating the parts to each other), intrinsic motivation and meaning orientation. On the other hand, a surface approach describes passive memorization, serialist style (dealing with the parts in isolation), extrinsic motivation and reproducing orientation [21, 22]. Each approach entails a combination of motive and strategy [23, 24]. Kember et al. [24] indicated that learners with deep motive may possess intrinsic interest and commitment to work, wherein learners who adopt deep strategies are more willing to connect ideas together and seek understanding. In contrast, surface motive learners may fear failure and merely aim for qualifications; learners who adopt surface strategies are more likely to minimize the learning scope and memorize materials.

Furthermore, researchers have recommended that students’ approaches to learning may be domain specific [24, 25]. Based on Kember et al.’s Revised Learning Process Questionnaire (R-LPQ-2 F), Lee and his colleagues validated a questionnaire to measure students’ approaches to learning science, namely the Approach to Learning Science Questionnaire (ALS). The constructs of the ALS contained four dimensions. While surface motive (i.e., extrinsic value) and surface strategy (i.e., rote learning) represented a surface approach, deep motive (i.e., intrinsic value) and deep strategy (i.e., meaningful learning) characterized a deep approach. Extended from Lee et al.’s ALS, researchers have also modified it to assess students’ approaches to learning in diverse domains such as biology [6] and physics [11].

With regard to the relationships between epistemic beliefs and learning approaches, Schommer [15] indicated that epistemic beliefs may have effects on students’ processing of information and monitoring of their comprehension. Hofer [1] found that sophisticated epistemic beliefs were correlated with intrinsic motivation (i.e., mastery orientation) and self-regulation strategies (i.e., metacognitive learning processes) but not significantly related to elaboration strategies (i.e., pulling information together). Tsai [26] reported that when learning science, students with more constructivist epistemic beliefs tended to employ more active and meaningful learning approaches; on the contrary, students holding empiricist epistemic beliefs were inclined to adopt rote learning approaches. Cano [4] found that there were positive relationships between sophisticated epistemic beliefs and a deep approach as well as positive correlations between naïve epistemic beliefs and a surface approach.

In relation to the domain-specific epistemic beliefs and their relations with learning approaches, Liang et al. [5] found that students’ beliefs about the development and justification of scientific knowledge can positively relate to their deep motive for learning science; in particular, it was reported that mixed motives (i.e., surface motive and deep motive) were positively correlated to sophisticated epistemic beliefs regarding justification. Lin et al. [6] reported that students who tended to justify epistemic assumptions and knowledge relating to biology had a stronger tendency to have mixed motives, and were more willing to utilize deep strategies to learn biology [6] Chiou et al. [11] found that there were associations among students’ beliefs about learning and knowledge and their approaches to learning physics [11].

Due to the domain-specific nature of epistemology, [7, 8] exploring the relationships between medical students’ medicine-specific epistemic beliefs and approaches to learning medicine may be contributive. By understanding the association of these two constructs, medical educators may appropriately modify medical students’ epistemic beliefs and alter their approaches to learning medicine. To this end, this study aimed to examine medical students’ medical-specific epistemic beliefs and their relationships with approaches to learning medicine including motives and strategies. To collect the research data, a questionnaire-based survey was employed; in addition, structural equation modeling (SEM) analysis was administered to test the structural relations.

**Fig. 1:**
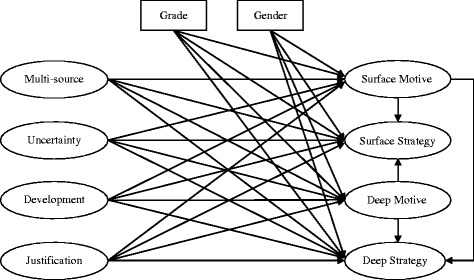
The hypothetical model of structural relationships between epistemic beliefs about medicine and approaches to learning medicine

The relations between motivation and strategies have also been discussed. For example, students’ motivational profiles and their relationships with reading strategies have been examined, and it was suggested that students with mastery goal orientation had more adaptive motivation and adopted more adequate strategies than those with a high work-avoidance goal orientation [27]. The positive relationships between surface motive and surface strategy as well as between deep motive and deep strategy have also been indicated in learning science [25]. Hofer [28] presumed that students’ epistemic beliefs about knowledge and knowing may affect their motivation, which in turn may influence their strategy selection and learning. Kizilgunes et al. [3] modeled the relationships among students’ epistemic beliefs, motivation, learning approach and achievement, and suggested that students need to develop sophisticated epistemic beliefs and meaningful learning. According to the aforementioned research, a hypothesized research model was constructed in this study and is displayed in Fig. 1. It can be proposed that medical students’ epistemic beliefs about medicine may be related to their motives for learning medicine; further, their learning strategies may be linked to such motives. However, mixed results of the relationships between goal orientation and learning strategies have been indicated. For example, it was revealed that performance-goal orientation may be linked to both surface strategy and deep strategy [3]. It was also found that students’ motivational orientations (learning, performance and multiple) did not result in differences in their use of deep learning strategies [29]. Therefore, it may be worth trying to identify the association between surface motive and deep strategy and the connection between deep motive and surface strategy. In Fig. 1, besides the relationships between epistemic beliefs and approaches to learning (motive and strategy), the correlations among motives and strategies are also examined. Since previous studies have reported these relations with combined results, the structural relations in Fig. 1 are indicated without specifying their positive or negative correlations. In addition, some studies have reported that there were differences regarding students’ approaches to learning between genders [30, 31]. Also, it was indicated that medical students’ approaches to learning may vary across their grade level during the medical program [12, 13]. Accordingly, the variables of medical students’ gender and grade level were simultaneously included in the hypothesized model to explore their influences on approaches to learning medicine.

## Method

### Participants

Nine medical colleges in Taiwan were targeted to conduct the survey. Each medical student from a seven-year medical training program before taking the licensing examinations for physicians was a potential participant. The representatives, teachers or students, recruited from nine medical colleges were requested to help with data collection. Through their support one of our research assistants went to each school to administer the survey. A total of 450 potential participants were contacted and asked to take the survey. Finally, 340 undergraduate medical students ranging from grade 1 to grade 7 completed the survey, with a response rate of 75.56 %. Their ages ranged from 17 to 32, with an average of 20.85. Since more males than females are enrolled in medical school in Taiwan [32], the majority of the participants were males (70.3 %), with a total of 239 males and 101 females, suggesting appropriate sampling of the participants.

### Instruments

Two Chinese version questionnaires, namely the Medical-Specific Epistemic Beliefs (MSEB) questionnaire and the Approaches to Learning Medicine (ALM) questionnaire, were administered to assess the medical students’ views on medical-specific epistemic beliefs and approaches to learning medicine. Both measurements were modified from previous studies in science [5, 11, 25]. To confirm their content validity, one expert in science education and another in medical education reviewed and made suggestions regarding the constructs and items of the instruments. A total of 25 items were selected in these two instruments, 12 for MSEB and 13 for ALM. All items of the MSEB and ALM were rated on a 7-point Likert scale ranging from 1 (strongly disagree) to 7 (strongly agree).

Based on Conley et al.’s [10] four-dimensional model of epistemic beliefs regarding science, the MSEB questionnaire was constructed with four factors, namely multi-source, uncertainty, development and justification. Further, the items of the MSEB were developed by modifying Lin et al.’s [6] and Liang et al.’s [5] questionnaires relating to epistemic beliefs in biology and science, respectively. The definitions and details of the MSEB questionnaire are described as follows:

*Multi-source*: assesses the beliefs that medical knowledge is not only retrieved from external authorities but is also generated from multiple sources and is constructed by oneself. The participants with sophisticated beliefs about multi-source tend to question the authority and believe that medical knowledge may come from multiple sources. An example item: Everyone has to believe what medical experts say (reversed item).

*Uncertainty*: evaluates the belief about whether there is only one right solution to a medical problem. The students with sophisticated views on uncertainty are inclined to think that there may be more than one solution to complex problems of medicine. An example: Medical knowledge is always true (reversed item).

*Development*: reflects the beliefs that medical knowledge is an evolving subject and can be changed on the basis of new evidence. The participants with mature beliefs about development hold views on changeable medical knowledge. An example item: Some medical ideas of the day are different from what medical experts used to think.

*Justification*: concerns about the ways in which students evidence and evaluate the medical claims. The participants with sophisticated beliefs about justification are willing to conduct experiments to support medical arguments. An example item: Doing experiments is a good way to justify if the medical ideas are true.

The ALM questionnaire was modified from Lee et al.’s [25] Approaches to Learning Science (ALS) questionnaire, which in turn was derived from Kember et al.’s [24] Revised Learning Process Questionnaire (R-LPQ-2 F), consisting of four factors labeled as surface motive, surface strategy, deep motive and deep strategy. The definitions and contents of the ALM questionnaire are illustrated as follows:

*Surface motive*: assesses the extent to which students hold extrinsic motives for learning medicine such as fearing failure in examinations and satisfying the expectations of their parents. An example item: I worry that my performance in medical class cannot fulfill the expectations of my teachers and parents.

*Surface strategy*: evaluates students’ inclination to utilize surface strategies for learning medicine such as narrowing targets and memorizing materials. An example item: Instead of understanding, memorizing important medical contents may help me get high scores in the examinations.

*Deep motive*: appraises the level to which students possess intrinsic motives for learning medicine such as having interest in studying medical issues. An example item: I always look forward to going to medical classes.

*Deep strategy*: estimates students’ intention to use deep strategies for learning medicine such as relating materials and maximizing understanding. An example item: When learning medicine, I try to find the correlations among the medical contents which I have learned.

### Data analysis

There were two stages of data analysis employed in this study, including confirmatory factor analysis and path analysis. The use of structural equation modeling technique (SEM) may allow researchers to estimate structural relations among variables and account for the measurement errors simultaneously. It was suggested that SEM has potential to advance research in medical education [33]. Therefore, this study performed SEM technique with 340 observations using AMOS 18.0 to examine the measurement model (i.e., confirmatory factor analysis for instruments) and structural model (i.e., path analysis for relations among variables). The SEM approach may evaluate the extent to which the collected data fit the hypothesized model. In general, maximum likelihood (ML) method was chosen for estimation.

First, the confirmatory factor analysis was executed to validate the two questionnaires. All items and factors of the MSEB and ALM were included in a single model of confirmatory factor analysis (CFA) to clarify the reliability and validity of both instruments. In addition, the values of factor loadings, average variance explained (AVE) and composite reliability (CR) were estimated to evaluate the validity and reliability of the measurement model. Next, using SEM, the path analysis was performed to test the relations between the epistemic beliefs about medicine and approaches to learning medicine in the structural model. Finally, according to the fit indices, the results of SEM in relation to path analysis may determine whether the hypothesized model is valid for explaining the structural relations among MSEB and ALM.

### Ethical consideration

This study was approved by the research ethics committee of National Taiwan University (NTU-REC No. 201505HS002). All of the participants included in this study voluntarily participated in the survey and responded to the questionnaires. The cover statement relating to the purposes of the study and participants’ rights was read before they answered the questionnaire. The participants were informed that they could refuse to take part in the survey, and if they did participate in the survey, their responses would be treated confidentially. Their permission signatures on the cover statement handed in together with the questionnaires were regarded as consent to participate in the survey.

## Results

According to the results of CFA, all items of MSEB and ALM have significant factor loadings ranging from 0.65 to 0.95, indicating suitable loadings which are not smaller than 0.5 or larger than 0.95 [34]. As presented in Table 1, the skew and kurtosis coefficients for items ranging from −0.96 to 0.14 are over −2 and less than 2, suggesting that no item has a severe normality problem [35]. Also, the composite reliability (CR) and the average variance extracted (AVE) were calculated to assess the reliability and convergent validity of the two measures. Using the threshold value of 0.6 as a criterion, the CR values range from 0.83 to 0.93, indicating acceptable reliability of the constructs; in addition, the AVE values ranging from 0.62 to 0.81 are higher than 0.5, showing the reasonable convergent validity of the constructs [34, 36]. The goodness of fit index (GFI) = 0.89 and adjusted goodness of fit index (AGFI) = 0.85 are not particularly high but still satisfy an acceptable value of 0.8 [37]. In addition, other model fit indices (*χ*^2^/ *df* = 2.29, normed fit index (NFI) = 0.93, comparative fit index (CFI) = 0.96, and root-mean-square error of approximation (RMSEA) = 0.062), suggest a reasonable data-model fit [38].

**Table 1:** Confirmatory factor analysis for the MSEB and ALM

	skew	kurtosis	λ	Mean	SD	CR	AVE
MS1: In medicine, only medical experts and professors know what is right.	−0.43	−0.51	0.91	4.61	1.41	0.89	0.74
MS2: In medical class, whatever the teacher says is true.	−0.43	−0.60	0.87				
MS3: Everyone has to believe what medical experts and professors say.	−0.29	−0.89	0.79				
UC1: Medical knowledge is always true.	−0.69	−0.56	0.95	4.87	1.57	0.93	0.81
UC2: Once medical experts get a result from an experiment, that is the only answer.	−0.69	−0.64	0.94				
UC3: The most important point of doing medicine is to come up with the right answer.	−0.40	−0.78	0.80				
DE1: Sometimes medical experts may change ideas which they thought were right in the past.	−0.96	−0.16	0.95	4.94	1.63	0.91	0.78
DE2: The ideas in medical textbooks sometimes change.	−0.77	−0.57	0.92				
DE3: Some ideas in medicine today are different from what medical experts used to think.	−0.53	−0.90	0.76				
JU1: It is good to have one’s own idea before starting an experiment.	−0.63	−0.54	0.80	4.89	1.49	0.90	0.75
JU2: Doing medical experiments is a good way to know if a medical idea is true.	−0.81	−0.35	0.94				
JU3: It is good to try experiments more than once to make sure if the finding is true.	−0.70	−0.51	0.85				
SM1: When I get a poor mark on a medical test, I worry about my performance on the licensing examinations.	−0.46	−0.92	0.80	4.59	1.45	0.89	0.74
SM2: Even if I have studied hard for a medical test, I still worry that I may not be able to do well on it.	−0.50	−0.70	0.95				
SM3: I worry that my performance in medical classes may not satisfy my teachers' and parents' expectations.	−0.46	−0.53	0.83				
SS1: When learning medicine, I try to memorize the content over and over until I remember it very well.	−0.33	−0.15	0.65	4.59	1.25	0.83	0.62
SS2: When learning medicine, I focus on and memorize the contents which may appear in examinations.	−0.52	−0.36	0.76				
SS3: When learning medicine, I use multiple ways of remembering to help my memory.	−0.65	−0.31	0.93				
DM1: I always look forward to going to medical class.	−0.24	−0.42	0.84	4.54	1.18	0.90	0.69
DM2: I spend a lot of my free time researching medical issues which have been discussed and I am interested in.	−0.24	−0.60	0.85				
DM3: I am satisfied with working on medical topics by myself to come up with my own conclusions.	−0.38	−0.39	0.86				
DM4: I learn medicine because of my enjoyment of studying medical issues.	−0.25	−0.64	0.77				
DS1: While learning medicine, I try to find the relationships among the contents which I have learned.	−0.71	−0.01	0.94	4.92	1.31	0.92	0.79
DS2: When learning medicine, I like to form theories to put odd things together.	−0.44	−0.16	0.81				
DS3: I try to understand the meaning of the contents which I have read in medical textbooks.	−0.88	0.14	0.92				

*Note*: *MS* multi-source, *UC* uncertainty, *DE* development, *SM* surface motive, *SS* surface strategy, *DM* deep motive, *DS* deep strategy, *λ* standard coefficients, *CR* composite reliability, *AVE* average variance extracted, *Mean* factor means (average score of factor items), *SD* standard deviations

**Fig. 2:**
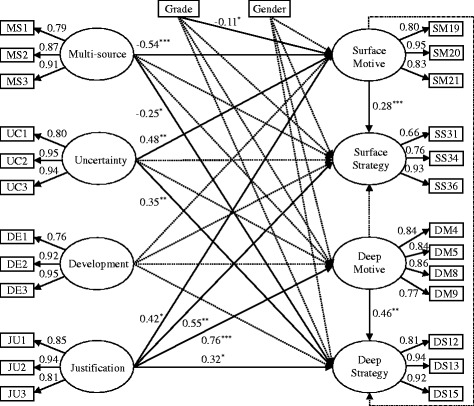
The results of the structural equation model regarding structural relationships between epistemic beliefs about medicine and approaches to learning medicine. *Note*: ^*^ *p* < 0.05; ^**^ *p* < 0.01; ^***^ *p* < 0.001; *solid lines* denote significant relations while *dotted lines* represent insignificant relations

The SEM combined structural model with measurement model is displayed in Fig. 2. The results of SEM showed adequate fit indices (*χ*^2^/ *df* = 2.25, normed fit index (NFI) = 0.92, comparative fit index (CFI) = 0.96, goodness of fit index (GFI) = 0.88, adjusted goodness of fit index (AGFI) = 0.84, and root-mean-square error of approximation (RMSEA) = 0.061), indicating a reasonable data-model fit. Table 2 shows the structural correlations between MSEB and ALM. Except development, other dimensions of MSEB have relationships with ALM.

**Table 2:** Structural correlations of SEM analysis

	Surface motive	Surface strategy	Deep motive	Deep strategy
Multi-source	−0.54^***^	−0.25	−0.003	−0.25^*^
Uncertainty	0.48^**^	0.19	−0.13	0.35^**^
Development	0.24	0.07	−0.06	0.19
Justification	0.42^*^	0.55^**^	0.76^***^	0.32^*^
Surface motive	NA	0.28^***^	NA	−0.06
Deep motive	NA	0.03	NA	0.46^***^

Note: ^*^ *p* < 0.05; ^**^ *p* < 0.01; ^***^ *p* < 0.001; NA: not applicable

As presented in Fig. 2, multi-source of medical knowledge is negatively correlated to surface motive (−0.54) and deep strategy (−0.25). Uncertainty of medical knowledge positively correlates to surface motive (0.48) and deep strategy (0.35). In addition, justification for medical knowledge is positively associated with surface motive (0.42), surface strategy (0.55), deep motive (0.76) and deep strategy (0.32). With respect to the approaches to learning, surface motive positively links to surface strategy (0.28) while deep motive positively connects with deep strategy (0.46). In addition, grade level has a negative relationship with surface motive (−0.11).

## Discussion

### Mixed motives for justification

According to the results of the path analysis, medical students who believed that medical knowledge comes from reasoning, thinking and experimenting tended to possess stronger performance-goal motives such as passing the certificated examination, and mastery-goal motives such as having passion for studying medical topics. Similarly, this phenomenon of mixed motives has also been found in various domains. Liang et al. [5] found that science-major undergraduates with sophisticated beliefs about justification of scientific knowledge were inclined to possess both surface and deep motives for learning science. In Lin et al.’s [6] study, it was also indicated that biology-major undergraduates’ epistemic beliefs of justification were positively correlated to both surface and deep motives. Furthermore, there were studies showing that the justification of knowledge is not only linked to a goal to understand and acquire competence (i.e., mastery-goal orientation) but is also unexpectedly linked to the performance-approach goal [39]. Ricco et al. [40] found consistent results indicating that students who regarded justification as necessary were likely to have mastery goals as well as performance goals. Researchers reported mixed findings for justification, and claimed that performance goals are not always maladaptive in all aspects [39, 40]. In the context of learning medicine, there could be an assumption that depending on learning contexts, both surface motives and deep motives can inspire medical students to make efforts to study medicine. In particular, based on the path coefficients presented in Fig. 2, either surface or deep motive has a positive influence on learning strategies, implying making efforts to learning medicine regardless of motives.

Another possible explanation for the mixed motive of justification is that succeeding in medical licensing examinations, an extrinsic motive, is an essential qualification for becoming a physician. It was reported that for Singaporean medical students, gaining knowledge for their future career was the most important objective to pursue in their medical education, followed by developing an ability to learn on their own [41]. Although intrinsic motives such as self-fulfillment and enjoyment can inspire medical students to make efforts in learning medicine, passing examinations and possessing a physician’s license to practice in medicine may encourage them to study hard to be a good doctor. To a certain extent, this may interpret why medical students with views regarding justification of medical knowledge were more likely to have stronger deep motives as well as stronger surface motives. Since they have strong intentions to justify the medical knowledge, they may have the ability to adjust various motives according to diverse learning demands and situations.

### Mixed strategies for justification

Besides mixed motives, epistemic beliefs of justification were also linked to mixed strategies. As shown in Fig. 2, it is indicated that those medical students who were inclined to justify medical knowledge were more likely to adopt not only surface strategies but also deep strategies. While discussing theories of medical knowledge in complex and uncertain clinical practice, Thomas [42] proposed that there should be three fundamental theories of medical knowledge, including objective evidence of already known diseases (positivism), evidence needed to be discovered and interconnected (critical theory), and subjective evidence which should be developed (constructivism). By using these three different theories of knowledge to listen, reflect and diagnose, physicians may practice well in complex, integrated and changing conditions [42]. According to this view, it can be supposed that medical students who hold views on justification of medical knowledge may believe in the complicated theories of medical knowledge and intend to undertake mixed strategies to memorize essential medical knowledge (surface strategy), correlate interconnected medical evidence (deep strategy) and construct new meanings of medical knowledge (deep strategy).

### Flexible approach for uncertainty

In general, it is supposed that surface motives should be accompanied by surface strategies [23, 24]. However, this study found that medical students holding views of uncertain medical knowledge tended to possess stronger surface motives and stronger intentions to adopt deep strategies, that is, they had a flexible approach. In other words, even though they were eager to pass examinations, they were inclined not to adopt rote learning for the reason that they believe in uncertain medical knowledge. Sturmberg and Martin [43] suggested that health care can be defined as a dynamic construct with multiple dimensions, which discriminate medical knowledge into the application of already known knowledge and the emergence of new meanings. They clarified that uncertainty is the inherent nature of medical knowledge, suggesting that learning medicine requires a context-driven flexible approach to applying already known medical knowledge and discovering new emerging medical knowledge [43].

Besides the deep approach represented by both understanding and intrinsic interest, Mattick, Knight [44] found that medical students have the desire to become good doctors and simultaneously have the intention to acquire vocation-related knowledge, the combination of such motivation is termed the vocational approach to learning. They suggested that medical students with a deep approach to learning intend to have inherent motivation based on academic interest and personal understanding, whereas those with a vocational approach tend to have intrinsic motivation which is not associated with understanding but is related to a fear of harming their patients. According to this opinion, there could be a speculation that medical students with views of uncertain medical knowledge tend to have surface motives (e.g., passing the license examination to become a doctor) rather than deep motives (e.g., working on medical topics); nevertheless, they are more likely to adopt deep learning strategies to entirely understand the medical knowledge since they are suspicious of certain medical knowledge and are eager to carefully study useful medicine which does not harm their patients.

### Dilemma of multi-source

The most interesting finding of this study is that beliefs of multi-source were negatively linked to surface motive and deep strategy. This implies that medical students who suspected the medical knowledge transmitted from authorities and experts tended to have low level surface motives and were less willing to adopt deep strategies. In other words, medical students’ trust in medical knowledge transmitted from authorities and existing in textbooks may support their devotion to deep learning. It was claimed that reliance on authority is supportive of learning [40]. That is to say, medical teachers should not overemphasize the doubt about essential knowledge and well-constructed medical evidence transmitted from authorities, especially for medical students who already hold highly suspicious views of authority.

This dilemma of source has also been previously discussed. Ricco et al. [40] proposed two explanations for the unexpected findings in the relationship between reliance on authority and mastery goals. One possible explanation is that only the absolutist acceptance of authority is associated with unavailing and immature motives, whereas evaluative trust in authority is associated with availing and mature motives. That is, acceptance of authority complying with critical and reflective views may correlate to adaptive motivations. The second explanation of the unexpected finding is that students with adaptive motivation perhaps possess the view that once a scientific finding appears in a textbook or teacher’s lecture, it must have been justified through appropriate processes and can be trusted as being correct. A comment was made that it is not always necessary and beneficial for students to hold questionable beliefs about knowledge, for example in some certain contexts in which knowledge is considered fixed and true for a long span of time. In this case, it would be useful for students to rely on authorities [45].

It has been assumed that accepting the knowledge of authorities and focusing on their intended messages seems to be the wiser strategy when in complex and challenging contexts [46]. Bråten and his colleagues [46] indicated that readers who appreciate the important contents of trustworthy sources may be involved in more cognitive thinking to understand the contents more deeply; on the contrary, when they believe that the knowledge is subjectively constructed by themselves rather than transmitted from objective sources, they may be too skeptical to recognize trustworthy knowledge from outside sources. This idea can also be inferred into complex and challenging contexts in learning medicine; that is to say, it is not beneficial for medical students to suspect medical knowledge in all situations. When facing unfamiliar medical problems, medical students need the ability to retrieve fundamental medical knowledge (i.e., rote learning) and a capability to apply those fundamentals (i.e., deep learning). To suppose that medical experts need to develop a new biomedical technique, in such a circumstance they can successfully use inventive knowledge to develop the modern technique only if the fundamental knowledge from authorities can be well understood and elaborated [47].

### The disappearance of development

According to this study, a relationship between beliefs in development and approaches to learning was not found. In Ricco et al.’s study [40], it was reported that scientific knowledge as developing appeared redundant since it did not significantly predict motivational factors. Also, Mason and her colleagues [39] pointed out that the development of scientific knowledge is not linked to motivational orientations. In line with these findings, it was indicated that the development of knowledge was not related to motivational orientations [48].

### Adaptive epistemology

Although epistemic beliefs are conventionally viewed as a continuum ranging from less mature (naïve level) to mature (sophisticated level) beliefs, and it is consistently consented that sophisticated beliefs are related to deep learning approaches [1, 2, 15], some recent studies on epistemic beliefs have produced controversial findings, indicating that sophisticated epistemic beliefs are not necessarily correlated to a deep learning approach and self-regulated learning [16, 49]. Pieschl et al. [16] hypothesized that the so-called “sophisticated” students may be adaptive learners who adjust their learning approaches to various task demands. Elby and Hammer [50] proposed that epistemic sophistication needs to be defined according to context. For example, they claimed that it would not be sophisticated for students to possess a tentative belief that the earth is round instead of flat; on the contrary, it would be sophisticated to hold tentative views about theories of mass extinction. It was also claimed that the terminology of sophisticated and naïve epistemic beliefs may be problematic since epistemic beliefs that are adaptive in a specific context may not necessarily be adaptive in other situations [46]. For example, Bråten, Strømsø [51] indicated that students holding adaptive epistemic beliefs in complex knowledge may gain deep text understandings from reading multiple conflicting texts, while those with less adaptive beliefs may benefit from reading the content of one single textbook.

Drawing from the results of this study, medical students with views on uncertainty and justification of medical knowledge seemed to have adaptive epistemic beliefs relating to medical knowledge and learning. That is, medical students may flexibly adopt either deep approaches or surface approaches or both to learn medicine according to their task demands and learning contexts. Even if they held a high level of epistemic beliefs about justification, they would alternatively choose learning strategies for particular contents. For example, they may undertake rote learning to memorize certain medical knowledge such as anatomy; on the other hand, they should administer deep strategies for learning complicated medicine such as diagnostics. Since the context of medical education is a learning environment consisting of multiple domains and sub-domains in terms of hard sciences (e.g., pharmacology and biochemistry) and soft sciences (e.g., sociology and psychology), medical students who possess varying epistemic beliefs across diverse domains and contexts may simultaneously have simplistic and advanced understandings of medical knowledge [52].

### Differences of motive across grade

As reported in Fig. 2, medical students at a high grade level were less likely to possess surface motive. Similarly, previous cohort studies indicated that compared to early-year (year 1 or year 2) medical students, later-year students (year 4 or year 5) tended to hold fewer surface approaches such as lack of purpose, unrelated memorizing, bounded syllabus, and fear of failure; in addition, there were no significant differences in deep learning approach across grade levels [12, 13]. It was indicated that medical students commonly fear failure during the basic science year, and the decrease in surface motive may result from the students’ maturation. Also, this difference can be explained by the changes in learning environment and learning tasks, which can be regarded as the contextual factors of approaches to learning [31]. Medical students at a high grade-level usually have to learn medical knowledge in complicated learning contexts and apply knowledge in clinical settings, which is more closely related to the reality of life than in school; as a result, they no longer engage in learning for fear of failure in examinations or extrinsic expectations from others.

### Limitations

This study has some limitations. First, there are many individual factors such as personality [53], social factors such as discipline, social identification [31] and cultural differences [40] which may have impacts on students’ approaches to learning; however, only the demographics including gender and grade as well as epistemic beliefs about medicine were included in the hypothesized model of this study. As a result, the hypothesized model could only have limited explanations for the differences in medical students’ approaches to learning medicine. Second, since the participants of this study were recruited from nine medical schools in Taiwan, there is a need to consider the effects of school-level on students’ approaches to learning and to use a multi-level analysis technique to investigate the effects. Due to the relatively small school-level sample size in this study, the contextual factors from school were not estimated via the utilization of the multi-level approach. Finally, since purposive sampling rather than random sampling was employed in this study, sampling bias may exist and generalization of the study results could be limited.

## Conclusions

Personal epistemology is regarded as a system of independent epistemic beliefs which may have a distinct effect on learning [15]. As Bråten, Strømsø [54] indicated, epistemic beliefs are regarded as important antecedents of students’ goal adoption. According to the findings of this study, it was shown that medical students’ epistemic beliefs regarding medical knowledge were independently and diversely linked to their approaches to learning medicine.

With regard to the epistemic beliefs about multi-source, the findings of this study indicate that medical students with doubts about medical knowledge transmitted from authorities or textbooks (i.e., sophisticated epistemic beliefs) were less likely to adopt both surface motives and deep strategies. This finding may respond to the argument claiming that sophisticated students do not always adopt a deep approach to learning and self-regulatory learning [49, 55], suggesting that instead of overly suspecting authorities, having respect for and appreciation of experts can support medical students in acquiring already known medical evidence and knowledge.

It seems that there is a need to develop medical students’ ability to perform medical experiments which may encourage them to appropriately administer both surface and deep approaches to learning medicine. While experimenting they need to recognize already known facts and utilize these facts to reconstruct emerging medical evidence. Doing medical experiments may offer a way to bring together multidimensional and dynamic knowledge to understand the overall health of patients [42, 43].

In general, educators expect students to learn for learning’s sake. It is supposed that students who seek external approval tend to learn in a rote way and not for the sake of learning [3]. However, this study shows that students with beliefs relating to uncertain medical knowledge are inclined to possess surface motives for learning medicine and are more willing to adopt deep learning strategies. This finding implies that students who learn for instrumental purposes (e.g., being keen to pass the licensing examination) may also simultaneously have intention to employ deep learning strategies.

In the end, physicians have to treat patients with holistic care by integrating their abilities of listening, reflecting and diagnosing. To develop skills in holistic care for patients, physicians and medical students are encouraged to be sophisticated in terms of fundamental medical knowledge and to be reflective in applying these fundamentals. That is to say, it is not necessary for medical students to possess sophisticated epistemic beliefs in medical knowledge, but to have an adaptive epistemology to adopt an adaptive approach to learning depending on the learning contents and context. In conclusion, medical students are expected to be flexible learners when learning complex and changing medicine.

## Abbreviations

AGFI, Adjusted Goodness of Fit Index; ALM, Approach to Learning Medicine; ALS, Approach to Learning Science Questionnaire; AVE, Average Variance Explained; CFA, Confirmatory Factor Analysis; CFI, Comparative Fit Index; CR, Composite Reliability; DE, Development; DM, Deep Motive; DS, Deep Strategy; GFI, Goodness of Fit Index; Mean, Factor Means; ML, Maximum Likelihood; MS, Multi-Source; MSEB, Medical-Specific Epistemic Beliefs; NFI, Normed Fit Index; R-LPQ-2 F, Revised Learning Process Questionnaire; RMSEA, Root-Mean-Square Error of Approximation; SD, Standard Deviations; SEM, Structural Equation Modeling; SM, Surface Motive; SS, Surface Strategy; UC, Uncertainty; λ, Standard Coefficients
